# Preclinical 4D-flow magnetic resonance phase contrast imaging of the murine aortic arch

**DOI:** 10.1371/journal.pone.0187596

**Published:** 2017-11-08

**Authors:** Moritz Braig, Jochen Leupold, Marius Menza, Maximilian Russe, Cheng-Wen Ko, Juergen Hennig, Dominik von Elverfeldt

**Affiliations:** 1 Department of Radiology, Medical Physics, Medical Center—University of Freiburg, Faculty of Medicine, University of Freiburg, Freiburg, Germany; 2 Department of Radiology, Radiology, Medical Center—University of Freiburg, Faculty of Medicine, University of Freiburg, Freiburg, Germany; 3 Department of Computer Science and Engineering, National Sun Yat-sen University, Kaohsiung, Taiwan; University of California San Diego, UNITED STATES

## Abstract

**Purpose:**

Cardiovascular diseases remain the number one death cause worldwide. Preclinical 4D flow phase contrast magnetic resonance imaging can provide substantial insights in the analysis of aortic pathophysiologies in various animal models. These insights may allow a better understanding of pathophysiologies, therapy monitoring, and can possibly be translated to humans. This study provides a framework to acquire the velocity field within the aortic arch. It analyses important flow values at different locations within the aortic arch. Imaging parameters with high temporal and spatial resolution are provided, that still allow combining this time-consuming method with other necessary imaging-protocols.

**Methods:**

A new setup was established where a prospectively gated 4D phase contrast sequence is combined with a highly sensitive cryogenic coil on a preclinical magnetic resonance scanner. The sequence was redesigned to maintain a close to steady state condition of the longitudinal magnetization and hence to overcome steady state artifacts. Imaging parameters were optimized to provide high spatial and temporal resolution. Pathline visualizations were generated from the acquired velocity data in order to display complex flow patterns.

**Results:**

Our setup allows data acquisition with at least two times the rate than that of previous publications based on Cartesian encoding, at an improved image quality. The “steady state” sequence reduces observed artifacts and provides uniform image intensity over the heart cycle. This made possible quantification of blood speed and wall shear stress (WSS) within the aorta and its branches. The highest velocities were observed in the ascending aorta with 137.5 ± 8 cm/s. Peak velocity values in the Brachiocephalic trunk were 57 ± 12 cm/s. Quantification showed that the peak flow occurs around 20 ms post R-wave in the ascending aorta. The highest mean axial wall shear stress was observed in the analysis plane between the left common carotid artery (LCCA) and the left subclavian artery. A stable image quality allows visualizing complex flow patterns by means of streamlines and for the first time, to the best of our knowledge, pathline visualizations from 4D flow MRI in mice.

**Conclusion:**

The described setup allows analyzing pathophysiologies in mouse models of cardiovascular diseases in the aorta and its branches with better image quality and higher spatial and temporal resolution than previous Cartesian publications. Pathlines provide an advanced analysis of complex flow patterns in the murine aorta. An imaging protocol is provided that offers the possibility to acquire the aortic arch at sufficiently high resolution in less than one hour. This allows the combination of the flow assessment with other multifunctional imaging protocols.

## Introduction

4D flow phase contrast MRI is a powerful tool to visualize complex flow patterns. It has given fundamental insight into the development of cardiovascular diseases [1]. Therefore, 4D-flow may also hold great potential in providing valuable information in preclinical MRI, especially in mice. Disease models using genetically modified mice allow a better understanding of disease progression as well as experimental therapy monitoring. Nonetheless preclinical 4D flow MRI is still under development and mostly 2D methods have been used, as three dimensional acquisitions are to date highly time consuming [2,3]. 2D phase contrast MRI can visualize flow but does not enable examination of complex flow patterns. Janiczek et al. [4] acquired high resolution 4D flow images with a spiral acquisition but focused on wall shear stress and did not provide any description of complex flow patterns through streamlines or pathlines. Later Bovenkamp et al. [5] first assessed flow patterns in mice and were able to prove the principal feasibility. However, their work yielded rather low, anisotropic resolution. In addition, image quality might have been compromised by phase variations through signal variation of the longitudinal magnetization, as the used sequence is interrupted during respiration of the animal. This leads to ghosting artifacts [6] and may restrict streamline quality thus limiting the analysis of complex flow. Espe et al. [7] use continuous RF-excitation. In their work they demonstrate the benefits of a nine-point motion encoding scheme particularly for strong gradients e.g. low VENCs but did not acquire 4D-flow data. The duration of an acquisition is a crucial parameter in 4D flow measurements, as heart beat changes are more prominent the longer the mouse resides in the bore, thus the error on the resulting velocities could be increased. Additionally, a reduction of scan time is favorable as animals may suffer from long narcosis and dehydration. This study strives to provide an improved setup for 4D flow measurements in the mouse aortic arch with a measurement time below one hour. Flow values and other parameters are quantified. Using streamlines and pathlines we aim to visualize the complex blood flow in the mouse aortic arch.

## Material and methods

### Data acquisition

This study was carried out in strict accordance with international recommendations and the guidelines of the local ethics committee. The protocol was approved by the responsible committee: (Ref: Regierungspraesidium Freiburg AZ:35–9185.81/G-14/91). All examinations were performed under isoflurane narcosis, animal physiology was continuously monitored throughout the experiment, and all efforts were made to minimize suffering. This manuscript adheres to the ARRIVE Guidelines for reporting animal research. A completed ARRIVE guidelines checklist is included in the supporting information labeled S1 Checklist.

Animals (n = 7; 13 ± 4 weeks) were ordered from a commercial breeder and housed in an in-house animal facility, examined under narcosis (1–2% isoflurane in O_2_ during spontaneous breathing). Temperature was monitored and maintained around 36.4–37°C throughout the measurement using a water circulation system. A pressure sensitive cushion was placed underneath the animal to detect respiration and ECG electrodes were connected to the front paws. Mice were placed head first in supine position in order to bring the heart close to the surface coil. Respiration was maintained at around 70 b/min by adjusting the anesthesia depth. Measured heart periods were between 100 ms and 145 ms. For the experiments the C57BL/6N strain was used, which is a common inbred strain for animal research without any genetic modifications.

For the preclinical in-vivo 4D flow phase contrast MR imaging we employed a Bruker BioSpec 70/20 USR system with a maximal gradient amplitude of 676 $\frac{mT}{m}$ and a slew rate of $4570\frac{mT}{m}/ms$ operated with Paravision 6.0.1. It was equipped with a two channel transmit and receive cryogenic (26 K helium cooled, Bruker) mouse head surface coil. This coil shows a factor of 3–5 improvement in SNR compared to conventional room temperature coils [8]. Physiological observation and sequence gating was conducted with an SA Instruments 1030 (Stony Brook, NY 11790) monitoring device. A respiration and ECG gated balanced four point (Hadamard scheme) 3D cine phase contrast sequence from the manufacturer (FLOWMAP, Bruker BioSpin Ettlingen, Germany) was used as a framework. Unlike commonly done in human phase contrast imaging the velocity encodings are acquired subsequently in different heartbeats in order to achieve a high temporal resolution. Therefore the repetition time (TR) corresponds to the temporal resolution. In this sequence the steady state of the longitudinal magnetization is interrupted during animal respiration in triggered experiments resulting in a blanking time of around ~200 ms. Artifacts arising from this interruption of the steady state can often be neglected in conventional preclinical MRI. However, severe artifacts are visible when the highly sensitive cryogenic coil is employed without averaging of acquired data. Therefore, the sequence was redesigned to maintain the magnetization as close as possible to a full steady state condition. This was ensured by implementing dummy scans during respiration. If an R-wave is detected (rising-edge) from the gating device the acquisition runs for a defined time (TR * number of cine frames) that is set by the user to about 85% of the duration of the RR interval of the animal. A blanking of the remaining 15% of the ECG cycle is necessary to clear the ECG from disturbing artifacts due to gradient switching and to account for a changing heartbeat during the measurement. Therefore, interruption of the steady state of the magnetization is nearly eliminated, a blanking time of 15% of the R-R-interval remains.

In order to be able to compare our measurement times to previous publications we compare the scan times for an ungated non cine acquisition, as these values are not dependent on the individual animal heart rate and respiration rate. For the Cartesian sequence used by Bovenkamp et al. [5] and our sequence they calculate according to:

Scantime(nogating)

$$TR\;*phase\;en{coding}_{1}*\;phase\;en{coding}_{2}*\;No.\;of\;flow\;encodings\;(4)*averages$$

We used calculated Shinnar–Le Roux excitation pulses in cranial-caudal direction that enable a sharp profile of the acquisition volume, allowing a reduced FOV compared to conventional sinc pulses. Read encoding is set in medial-lateral direction and phase encoding dorsal to ventral. A very narrow FOV in phase encoding direction was used that causes slight folds into the image but not into the region of interest. This very narrow FOV was chosen in order to maximize resolution for a given acquisition matrix and therefore reduce scan time. A flip angle of 12° was empirically chosen under two considerations; 1) to achieve a high blood to tissue T_1_ contrast 2) in order to compensate for the non-homogenous excitation of the cryogenic coil, where the RF deposition drops sharply with increasing distance from the coil. The high blood to tissue contrast facilitates data post-processing as for example during isosurface generation of the aorta.

The sequence was validated on a phantom with the same VENC settings as in the in-vivo protocol and an ECG simulator in order to simulate a situation that resembles the in-vivo case was used. The phantom consisted of a tube with a diameter of 6 mm with water pumped through (non pulsatile steady flow). The mean velocity and volumetric flow rate through the tube were calculated by measuring the amount of water passing through the tube during unit time. In the acquired 3D MRI images the tube was segmented in the middle slice and flow and mean velocity through this ROI were calculated from the MRI velocity data.

### Processing

The raw data was reconstructed using Matlab (MATLAB 2015b, The MathWorks, Inc., Natick, Massachusetts, United States). All four velocity encodings and coil elements were combined together to produce the final sum-of-squares magnitude image. The four velocity images were combined using the Hadamard scheme. Noise masks were obtained from the magnitude data and applied to both velocity and magnitude datasets. For the noise masking the magnitude data was decomposed with a multilevel 3D wavelet decomposition (5^th^ order Daubechies wavelet with a decomposition level of four). Noise was then removed by a user defined threshold.

All data was corrected for filter delays arising from the ECG peak detection and ECG-signal-filtering. A user set threshold defines static tissue over the image, which is used for a second order phase offset correction as commonly used in human MRI [9].

SNR values were measured by taking the mean signal intensity of a ROI in the aorta (between the truncus brachiocephalicus and the branch of the left common carotid artery) and the standard deviation within an ROI placed in a region containing only noise (ventral) for the first timeframe of the magnitude image.

The measured SNR is taken from the magnitude image. It should be noted that the signal of the calculated magnitude image is the sum of all four encoding whereas the velocity images are a combination (Hadamard scheme) of all four encodings. SNR is spatially dependent as a surface coil was used. Thus we choose the aortic arch for the SNR measurements, which is the main region of interest. Flow and wall shear stress (WSS) analysis is described in [10] in detail. In short: the analysis planes (chosen perpendicular to the aorta) were segmented using B-splines. The WSS vector was separated into axial and circumferential components as explained in [10]. Values were evaluated at four planes perpendicular to the aortic arch. The aorta was manually delineated in each plane.

We adapted visualization and analysis techniques that are common in human flow analysis [9] for our data visualization and quantification. Ensight (Ensight, CEI, Apex, NC, United States) was used for data visualization. First a speed-sum-of-squares isosurface I_speed_ of the velocities was generated calculated by $I_{speed}=\sqrt{v_{x}^{2}+v_{y}^{2}+v_{z}^{2}}$. Only velocities within that isosurface were used for streamline and pathline generation for the masked visualization. In order to assess streamline and pathline quality we provide unmasked streamlines and pathlines. For a quantitative analysis of streamline quality we counted the number of unphysiological pathlines, similar to the approach described in [11]. Unphysiological streamlines are those streamlines that leave the isosurface or those that cannot be attributed to a normal animal physiology. An error on the number of streamlines was estimated (based on multiple counting from the same observer, were the maximum deviation from the mean was taken as error) as some streamlines could not clearly be attributed to be physiological or non-physiological. Streamlines were generated for each time frame individually from four emitter planes placed perpendicular to the aortic isosurface. These streamlines are instantaneous tangents to the velocity vector calculated individually for every cine frame of the heart cycle and that are emitted from cut planes perpendicular to the aorta at specified locations. So called pathlines were used to visualize flow over the heart cycle. A pathline is the line followed by an imaginary particle over time. In our case pathlines are emitted from two emitter planes at the beginning of systole, calculated over the whole heart cycle using the velocity information of all time points. This is different to streamlines where each time point is calculated separately without using the information from other time points. Both pathlines and streamlines are color coded with the magnitude of the velocity vector. Pathlines are emitted from two emitter planes with 300 emitters from each plane, restricted to the speed-sum-of-squares isosurface of the dataset. The streamline visualizations utilize 200 emitters from each plane.

Sequence parameters were adjusted under two propositions; 1) achieve a high temporal resolution (short TR below 6 ms) 2) accomplish the acquisition below one hour. The first was achieved by reducing the echo time by means of a partial echo in read direction and a bandwidth of 89286 Hz. Zero-filling was used for the reconstruction of the partial echo. Other sequence protocol parameters for the measurements are shown in Table 1.

**Table 1:** Protocol parameters used for the acquisitions.

	In-vivo parameters
Field of view	20 x 16 x 14 mm^3^,
Matrix	68 x 50 x 50
Resolution	300 x 320 x 280 μm^3^
Reconstructed resolution	160 x 160 x 160 μm^3^
Flip angle	12°
TE/TR (temporal resolution)	2/5 ms
Bandwidth	89286 Hz
Echo position	26%
No of averages	1
Phase offset correction	2^nd^ order
VENC	170 cm/s
Scan time in-vivo	~ 40 min
Scan time (no gating)	0.83 min

## Results

### Sequence improvements

**Fig 1:**
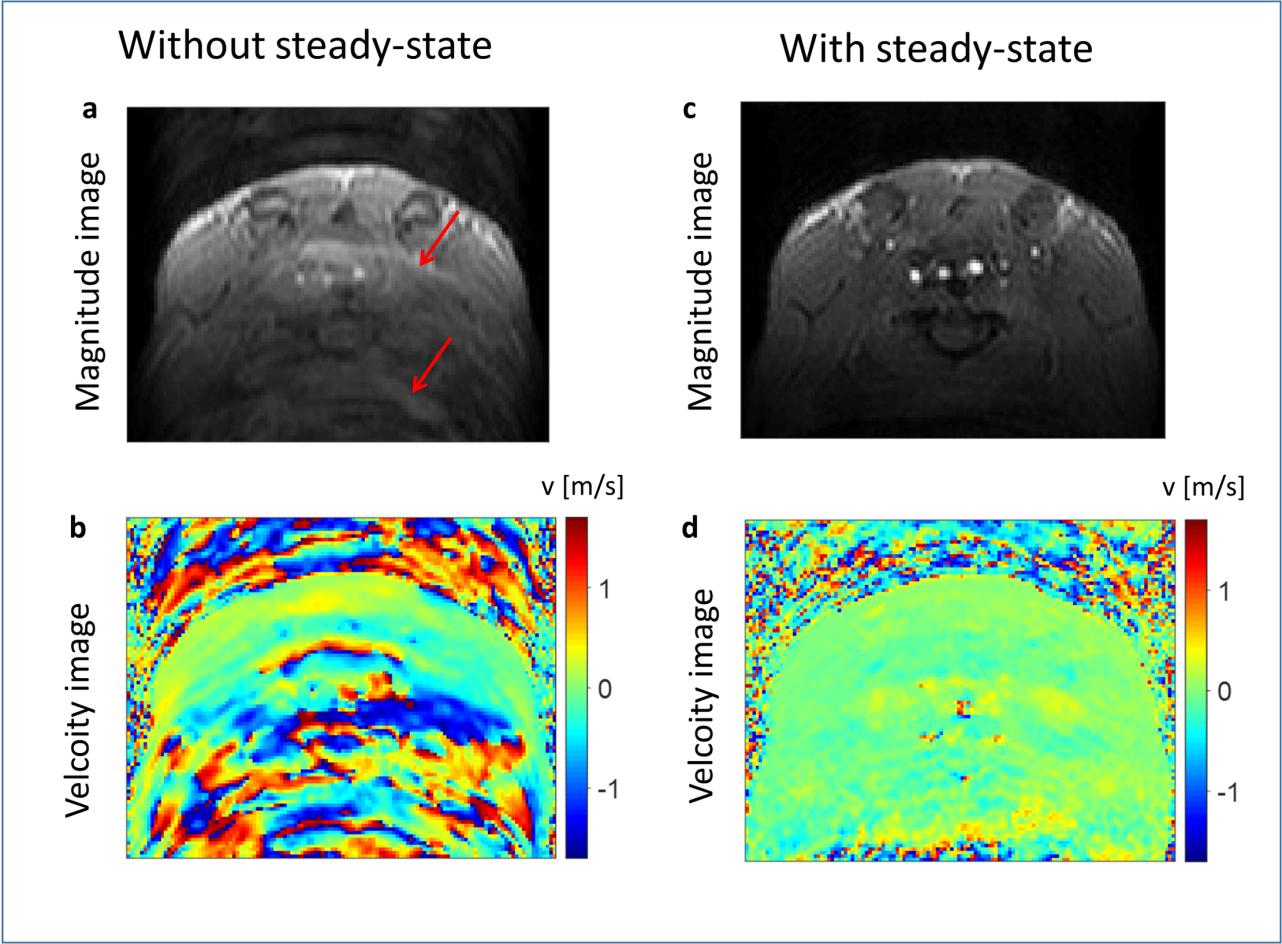
Comparison of the image quality before and after the sequence modifications. An axial in-vivo image of the upper mouse abdomen before (left side) and after sequence modifications (right side) is shown. Images a and c show the magnitude reconstruction of the acquisitions, b and d the corresponding flow images for one direction. One can see multiple ghosts (exemplarily shown with red arrows) in the phase encoding direction (top to bottom) in the images of the unmodified sequence (left side). These artifacts are removed when using dummy scans during respiration (right side).

Fig 1 shows axial magnitude and phase images of the upper mouse body before and after the implementation of dummy scans during respiration in-vivo. Ghosting artifacts are visible in the phase encoding direction of the non-steady-state acquisitions. These artifacts diminish towards later time frames. No relevant ghost artifacts are visible in the improved steady state acquisitions. Both acquisitions were acquired with the same respiration gating in the same mouse. A time resolved movie from the magnitude data of the improved sequence is available as supplementary material S1 Fig. The whole dataset was acquired in less than one hour.

### Analysis

#### Sequence validation

The flow verification on a tube phantom yielded a mean velocity of 11.6 cm/s and volumetric flow rate of 30.303 cm^3^/s from the MRI data compared to a mean velocity of 10.8 cm/s and a volumetric flow rate of 30.667 cm^3^/s from the measurement of water volume over time.

#### Flow and wall shear stress analysis

**Fig 2:**
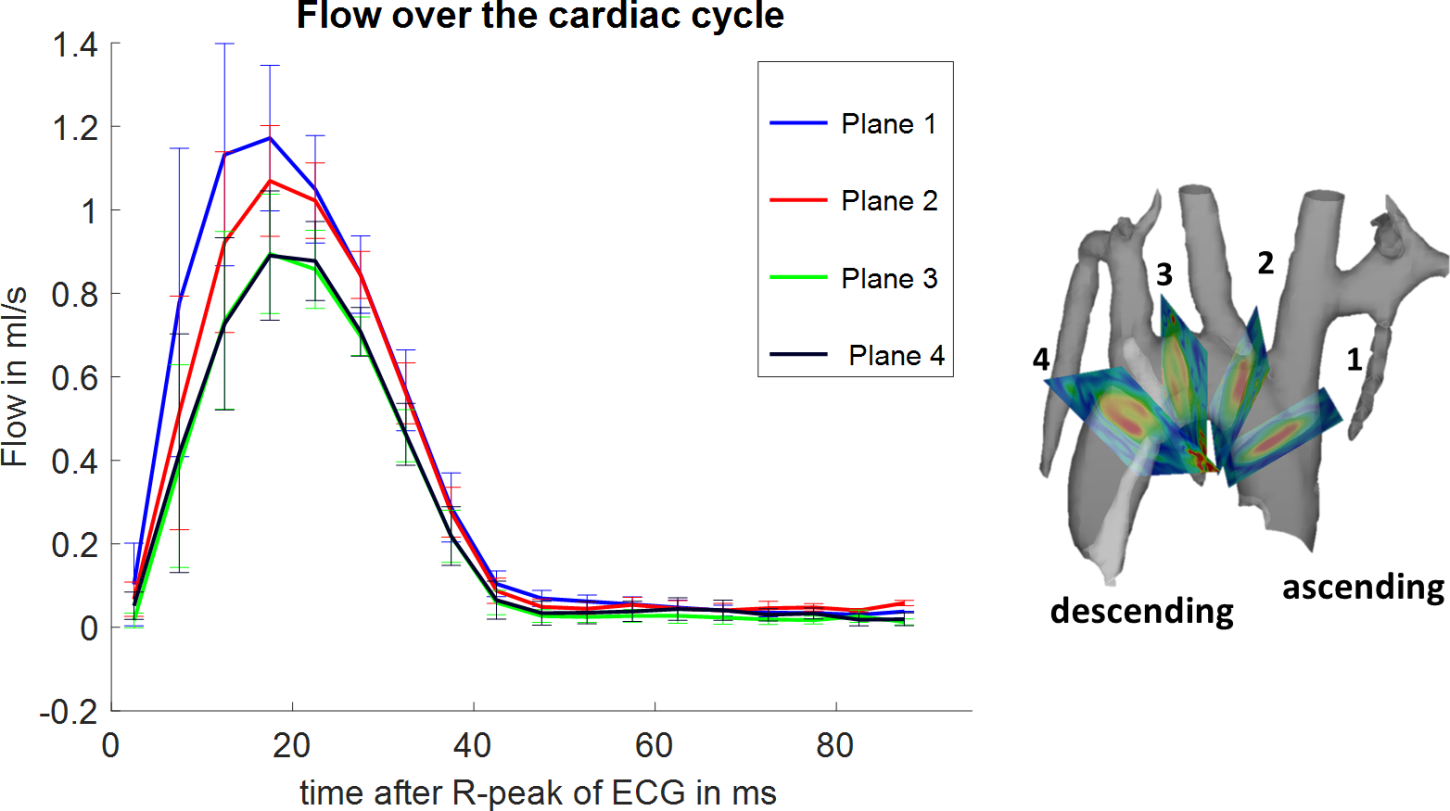
Mean flow over the cardiac cycle. Flow over the cardiac cycle for four evaluation planes placed at specified locations is shown on the right. Flow is highest in the ascending aorta (AAo). The curves show the mean value of all subjects and their standard error of the mean as error bars. One can observe a trend towards lower flow velocities (plane 1 towards plane 4) as expected and longer time to peak flow durations.

**Fig 3:**
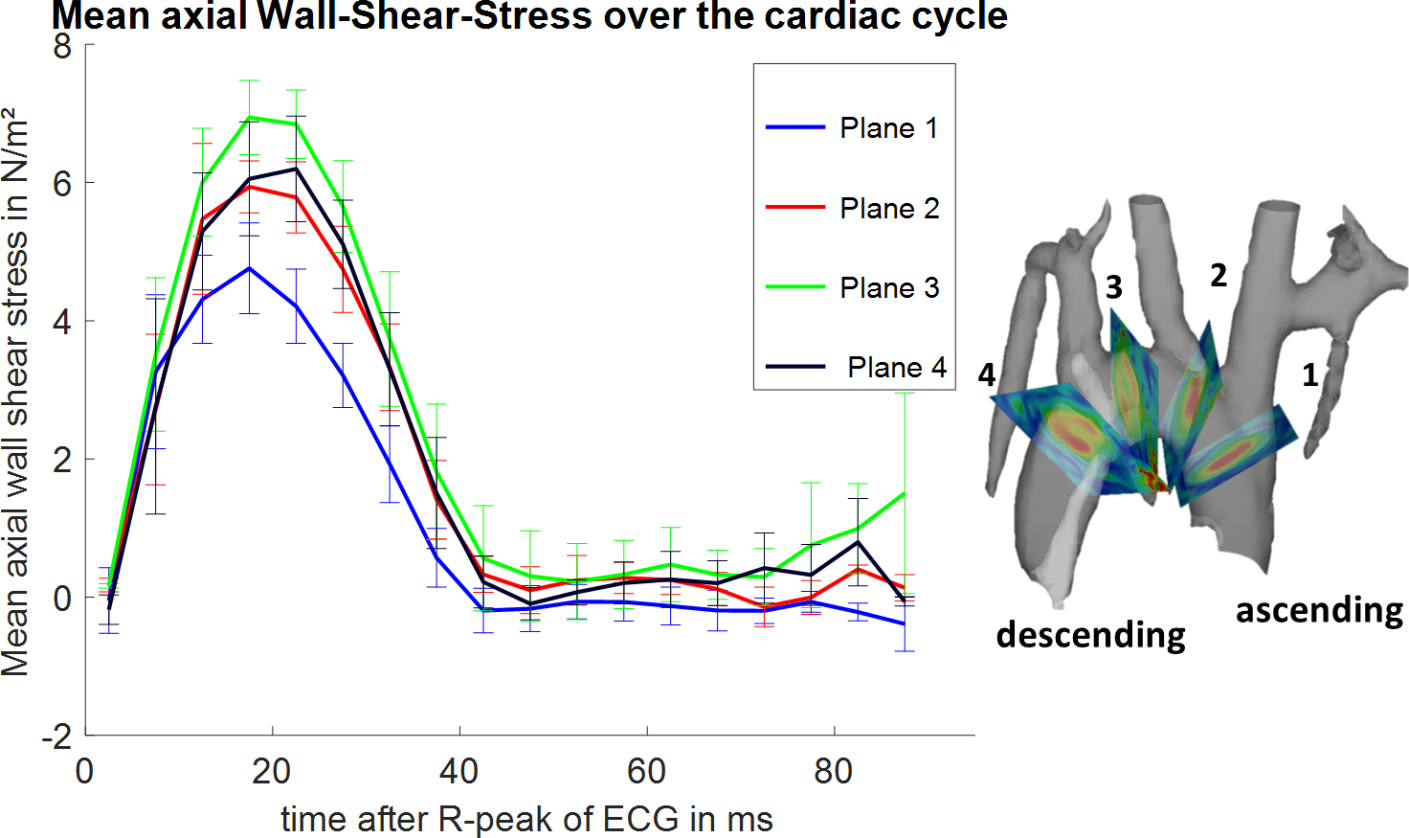
Mean axial wall shear stress over the cardiac cycle. Mean axial wall shear stress over the cardiac cycle for four evaluation planes placed at specified locations is shown on the right. The highest wall shear stress can be observed in the third plane. The curves show the mean value of all subjects and their standard error of the mean as error bars. The lowest mean axial wall shear stress occurs in the ascending aorta.

Flow through the analysed planes and wall shear stress was evaluated over the cardiac cycle for the four evaluation planes as shown in Figs 2 and 3. Fig 2 depicts the mean (all subjects) positive flow volume pixel wise through-plane for each plane individually within the segmented ROI. Plane 1 shows the highest peak-flow about 20 ms after the R-peak of the ECG. A trend towards longer time to peak flow volume values from plane 1 (ascending aorta) towards plane 4 (descending aorta) can be observed as well as a decrease in the maximum flow and total flow over time. Fig 3 shows the mean axial wall shear stress over the cardiac cycle as mean value of all subjects evaluated at four planes perpendicular to the aorta. Plane 1 shows the lowest mean axial wall shear stress; plane 3 shows the highest mean axial wall shear stress.

**Fig 4:**
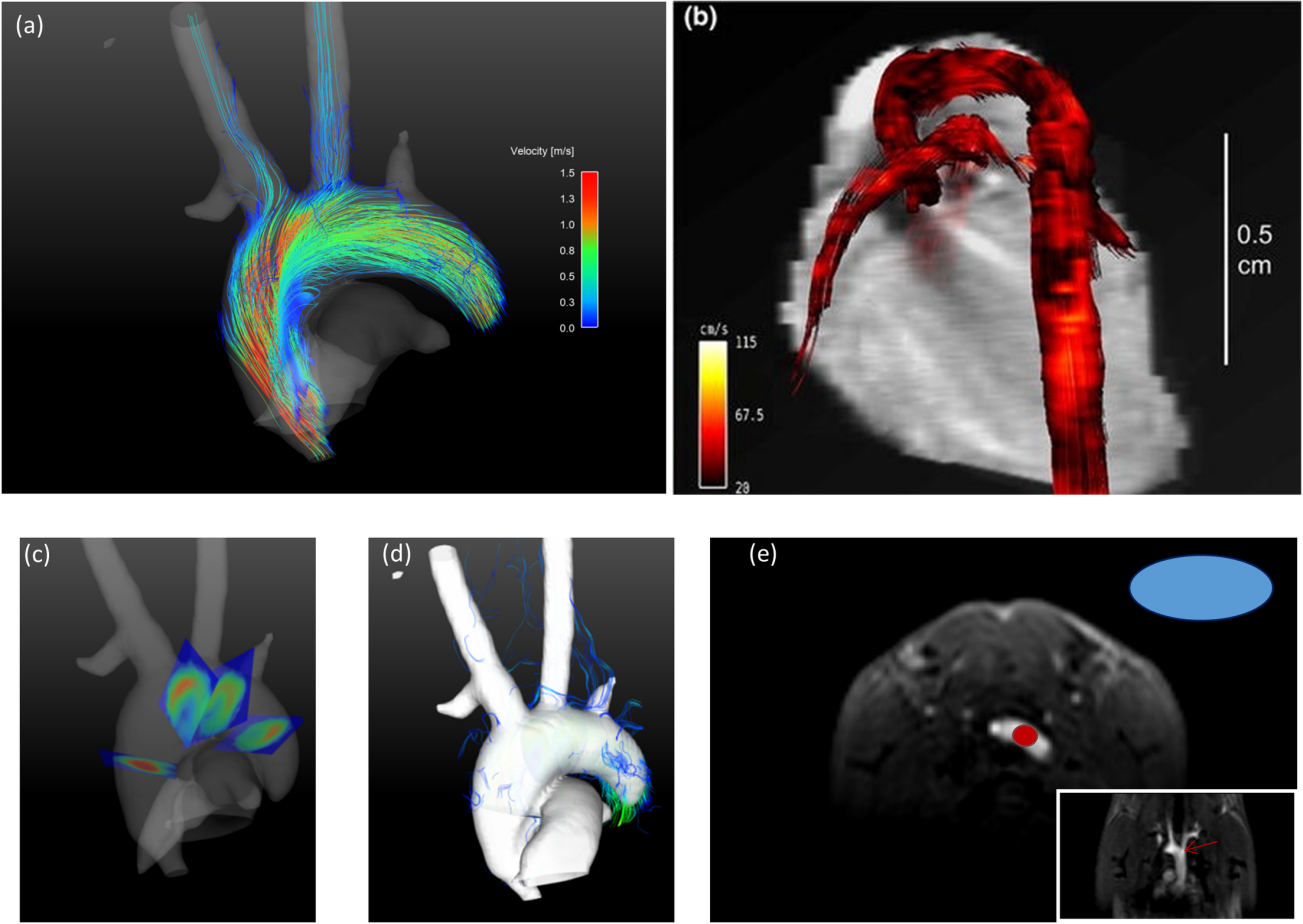
Streamline representation of the blood velocities during systole. a) Streamline representation of the velocities of one mouse during peak systole. Streamlines are generated from four emitter planes placed perpendicular to the aorta as shown in (c). Streamlines are color coded with the magnitude of the velocity as displayed in the color bar. The isosurface in this image was calculated as speed-sum-of-squares isosurface from the velocity data. One can identify regions of high velocities and low velocities. The vessel lumen is equally filled. Streamlines continue even in the small branches of the aorta. Velocities are lower in the aortic branches than in the aortic root. Supplementary animation S2 Fig provides extended streamline visualization from multiple viewing angles. b) Streamline representation of the velocities of one mouse during peak systole from Bovenkamp et al [5] figure 6 (clipped). *“Blood flow visualization with vector magnitude-encoded streamlines in the cardiovascular system of a mouse at 13*.*76 ms after R wave of the ECG”*. c)Visualization of the used emitter planes for the streamlines in the aortic arch. d)Unmasked streamlines are shown that leave the isosurface. The isosurface is shown opaque in order to easily identify aberrant streamlines. e)Placement of the ROIs for SNR analysis.

Peak velocities were 137.5 ± 8 cm/s, measured in the ascending aorta, 57 ± 12 cm/s in the brachiocephalic trunk and 42 ± 5 cm/s in the left common carotid artery (LCCA). The measured signal to noise ratio in the aortic arch in the magnitude images was 120 ± 12 over all measured subjects evaluated at the location shown in Fig 4E.

### Streamline and pathline visualization

Fig 4A depicts a streamline representation of the velocity data during systole. Streamlines are emitted from four emitter planes placed perpendicular to the vessel at different locations as shown in Fig 4C. The visualization is shown together with an isosurface generated as speed sum of squares mask of the velocities over time. This facilitates observation of the blood flow and identification of the anatomic region. Streamlines are masked by a speed-sum-of-squares isosurface. The streamlines originate from the emitter planes and can be evaluated even in the sub branches of the aorta. The dependence of the velocity on the position is clearly visible and velocities are easily distinguished. The highest velocities occur within the aorta; peak velocities in the branches are lower. A 3D visualization of the streamlines during systole from different viewing angles is available as supplementary material S2 Fig. Fig 4B is used for a side by side comparison to the streamlines visualization of Bovenkamp et al [5] with our result Fig 4A. A clip of figure 6 of their publication is shown. In Fig 4C the emitter planes where the streamlines originate are displayed and Fig 4D shows unmasked streamlines of the same time point in the cardiac cycle together with an opaque isosurface to easily identify aberrant streamlines. The number of non-physiological streamlines were counted to 220 ± 30 (27.5%), were 30 is an estimated error given the fact that some streamlines could not clearly be attributed to be physiological or non-physiological.

**Fig 5:**
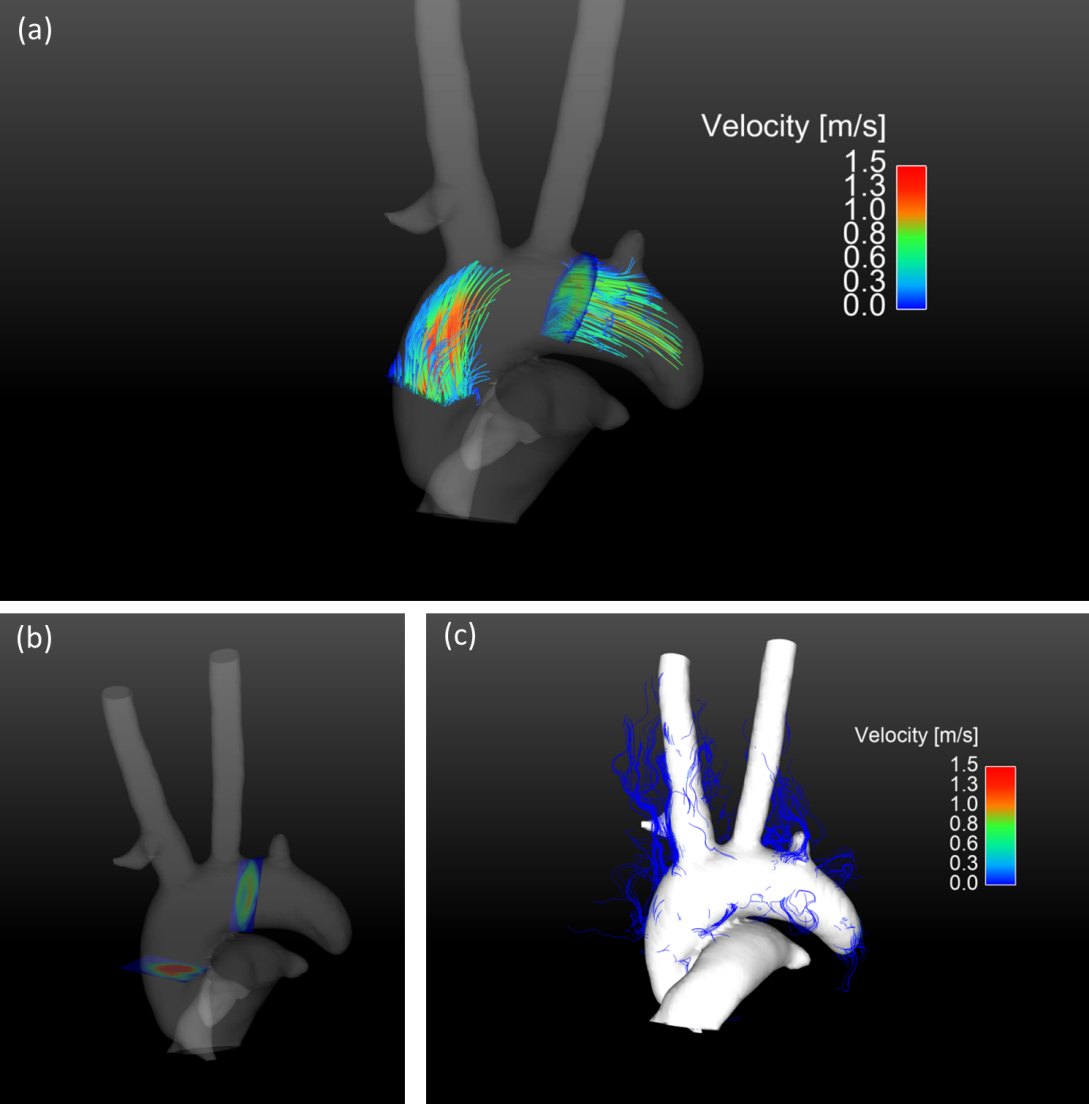
Pathline visualization of the blood flow in the aorta during systole. a) Pathline visualization during systole generated in early systole from two emitter planes perpendicular to the vessel. The emitted pathlines continue into the brachiocephalic trunk, left common carotid artery and the left subclavian artery. The pathlines are masked by a speed-sum-of-squares isosurface. A pathline animation over the cardiac cycle is shown in the supplementary animation S3 Fig. b) Visualization of the used emitter planes for the pathlines in the aortic arch. c) Unmasked pathlines are shown that leave the isosurface. The isosurface is shown opaque to easily identify aberrant pathlines.

Fig 5A shows pathlines during late systole generated from two emitter planes perpendicular to the vessel during early systole. A segmented speed-sum-of-squares isosurface enfolds the pathlines. The color bar represents the occurring velocities. A time resolved 3D visualization of the pathlines from different viewing angles is available as supplementary material S3 Fig. Pathlines can be traced up to the sub-branches of the aorta. Fig 5B provides the anatomical reference of the used emitter planes and Fig 5C shows the pathline visualization with unmasked velocity data.

## Discussion

This study provides a quantitative analysis of aortic flow and wall shear stress at several locations of the aortic arch in mice. The combination of a high SNR cryogenic coil and continuous RF-excitation allowed the acquisition of 4D velocity data that even can be evaluated in the branches of the aortic arch. Due to the high SNR and stable phase of the data, streamline visualizations for the blood flow both at high and low velocities are provided that continue up to the aortic branches. To the best of our knowledge this study provides the first pathline visualization of blood flow in the mouse aortic arch.

The benefits of a cryogenic probe in preclinical anatomical cardiovascular imaging using retrospective acquisitions have been shown previously [8]. Our research transfers these advantages to prospective preclinical 4D phase contrast flow measurements where long scan times, low resolution and low SNR have been a major drawback. The results show that it is not possible to use the cryogenic surface coil with the conventional sequence design for phase contrast imaging. Velocities are falsified due to ghost artifacts that arise from a non-steady state longitudinal magnetization. These artifacts occur as different k-space lines are acquired with different signal amplitudes. The same artifacts are also observed in stationary phantoms in gated acquisitions, where the sequence is interrupted in the same way as it would be in-vivo [12]. It is not possible to predict the quantity and intensity of these artifacts as they depend on the heart and respiration rate of the animal. The artifacts diminish towards later timeframes as the signal variations between the different k-space lines decrease while the longitudinal magnetization converges towards a steady state. After our sequence modifications these artifacts are negligible as sequence blanking is reduced to a value below 20 ms. These artifacts have already been described for magnitude images in preclinical imaging [6]. They may be negligible when using averaged magnitude or phase images; however in prospective phase contrast imaging without averaging they cannot be neglected. By implementing dummy scans during respiration, sequence blanking, and therefore the steady state artifacts can be minimized as shown in Fig 1.

Janiczek et al. [4] and Bovenkamp et al. [5] both reported 4D flow measurements in the murine aorta. Janiczek et al. employed a high resolution spiral acquisition (170 μm isotropic) providing a widespread analysis of wall shear stress. Unfortunately, no streamline or pathline representation is given. The first streamline visualization from 4D flow MRI in mice was shown by Bovenkamp et al. [5], in figure 6. Therein, calculated streamlines for every acquired time point of the cardiac cycle are shown. A quantitative comparison to those streamlines is not possible as the data is not available. As an alternative we analyze the differences in image quality qualitatively.

In Fig 4A and 4B streamlines of Bovenkamp et al. [5] and our measurements are compared. Despite the shorter measurement time of our acquisition, streamlines continue even into the branches of the aorta. This improvement in quality is likely due to an improved stability of the data due to the continuous RF-excitation and a more isotropic resolution. The streamlines of Bovenkamp et al. are cropped for velocities below 28 cm/s; this could explain why the streamlines shown do not completely reflect the shape of the aorta. It would be interesting to compare non-cropped data, as lower velocities are more sensitive to artifacts as the phase error increases for low velocities. We assessed streamline quality using data without restricting the velocity to the isosurface (Fig 4D). Unphysiological streamlines can be observed, but these are mainly streamlines with very low velocities where the phase error is high. In the descending aorta streamlines leave the isosurface. This can be explained by a signal drop towards the distal part of the mouse body where the signal-to-noise-ratio is low and therefore a generation of a continuous isosurface is not possible anymore. However, these streamlines are still physiological.

Spatial resolution in the measurements of Bovenkamp et al. is 75 x 280 x 670 μm^3^. Highly anisotropic and low resolution in one direction can be difficult when displaying complex flow in the curved aorta as partial volume effects can falsify measured velocities [13]. We believe that our higher, more isotropic resolution with superior SNR is preferential to display complex flow; especially as the diameter of the aortic arch is in the range of 1–2 mm in mice [14]. Previous publications have shown that a minimum of 3–4 pixels are necessary to measure accurate peak velocities [15]. Considering the smaller parts of the aorta, e.g. the descending aorta with mean diameter around 0.74 mm [16] our acquired resolution of about 300 μm results in 2.5 pixels per diameter and therefore less accurate velocity quantification for smaller vessels. In our experience accurate peak velocities can be obtained even in cases of low SNR, slight miss-triggering or even when minor artifacts are present. Streamlines and pathlines however can easily be interrupted by minor artifacts as they are dependent on stable image quality over the whole analyzed region. Our setup provides a good compromise between the necessary resolution and signal to noise ratio, while still allowing for the calculation of streamline and pathline representations.

Our measurements are two times faster at equal encoding steps compared with [5], as no averaging is needed. Observed in-vivo measurement times for our subjects were around 40 minutes, resulting in about one hour of total measurement time including scanner adjustments and mouse setup. The setup in [5] would need more than two hours as more phase encodings are used and two averages are employed.

Another major issue in preclinical cardiac MRI is the temporal resolution. In preclinical cardiac MRI the heartbeat is very short compared to the repetition time of the acquisition. Thus a high time resolution is necessary to resolve flow changes during the R-R cycle. It was possible to reduce repetition time to 5 ms compared to 6.9 ms in previous publications using Cartesian sampling [5], which is an increase of 30% in temporal resolution. This was achieved by using a partial echo readout of 26%. One has to note here that this partial echo readout reduces the acquired resolution in readout direction and lowers the signal to noise ratio.

In this work we present, to the best of our knowledge, for the first time pathlines from 4D flow MRI in mice. The pathlines shown in Fig 5A and supplementary video S3 Fig show the blood flow exemplarily for one mouse. Pathlines could provide additional information in pulsatile flow [17] as in presence of plaques or in complex congenital heart diseases and collateral vessels [13]. Our proposed setup might, for example, yield new insights in apolipoprotein E–deficient (apoE−/−) mice. These mice spontaneously develop atherosclerotic lesions on a standard chow diet and therefore show altered flow patterns. MRI 4D-flow imaging may provide understanding of flow changes and relation of these changes to plaque development and progression. Quantitatively derived values from the velocity information as wall shear stress are known to be related to plaque formation and pathlines are able to expose abnormal flow changes that may promote plaque progression.

In order to further improve the streamline and pathlines visualization, especially for very low velocities, it would be beneficial to have a higher resolution and signal to noise ratio or a dual VENC approach. One could possibly achieve even higher resolution and higher SNR by using intravascular contrast agents and decrease measurement times using acceleration techniques such as k-t-Grappa or compressed sensing. However, in our case the acceleration is limited as only two receiver coils are available for parallel imaging. In order to visualize the whole cardiac cycle a retrospective approach would be necessary, as the current prospective approach is limited to about 70–80% of the cardiac cycle. This restricts this method to the analysis of systole and diastole omitting the late diastole.

This study provides evaluated flow and wall shear stress at four different locations in the aortic arch. Peak flow values decrease towards the descending aorta and are highest in the ascending aorta (Fig 2) around 20 ms after the R-wave of the ECG. Time to peak flow values are shortest in the first analysis plane and occur later in the descending aorta. Analysis plane 3 and 4 show little difference in peak flow and time to peak values, whereas analysis plane 1 shows a much higher peak flow of around 1.2 ml/s and a short time to peak. Calculated wall shear stress values show a different behavior. Time to peak wall shear stress occurs tends to occur later in the cardiac cycle than time to peak flow values. The highest wall shear stress is observed in plane 3 (Fig 3).

The MR-protocol described in this study was adapted to analyze blood flow within the aortic arch, as this is one of the major regions of interest in preclinical cardiovascular research. The protocol could be modified to other regions; however it would need readjustment. A tradeoff between the necessary resolution for the assessed vessel size, signal to noise ratio, FOV and scan time must be individually considered for each application.

## Conclusion

Our improved setup enables the visualization of complex blood flow in the murine aorta and even its branches. By using a cryogenic coil and continuous RF-excitation, it is possible to acquire a high-resolution dataset showing complex aortic blood flow in less than one hour. These data can then be used to create streamlines of the velocity field in the aorta and its major branches that show a distinct better quality than previous publications with respect to phase errors and depiction of smaller vessels. Due to this short acquisition time, sufficient time is available to combine the blood flow analysis with other imaging protocols within one examination and to perform a multifunctional analysis for example with targeted contrast agents. In this work we provide flow values as well as an analysis of the occurring wall shear stress and complex flow patterns. To the best of our knowledge this is the first pathline visualization of the blood flow in the murine aorta. This setup provides the framework to study and quantify complex blood flow and hence assess various pathophysiologies in mice.

## Supporting information

S1 Checklist The ARRIVE guidelines checklist. Completed “The ARRIVE Guidelines Checklist” for reporting animal data in this manuscript.
(PDF)

S1 Fig Sample magnitude movie of one dataset. Sample video showing the beating mouse heart in oblique slice rendering from the acquired data.
(AVI)

S2 Fig Streamline visualization of the aortic blood flow. 3D rendering of the streamline visualization within the aortic arch during systole. Velocities are color coded according to the displayed scale. An isosurface generated as speed-sum-of-squares mask is used to enclose the velocity data. Streamlines are emitted from three emitter planes placed perpendicular within the aorta.
(MPG)

S3 Fig Pathlines visualization of the aortic blood flow. Pathline rendering of the aorta in 3D. Pathlines are emitted during beginning of systole and then calculated over the whole heart cycle. An isosurface created from the magnitude images is provided as anatomical envelope for every time frame separately in order to account for the changing geometry of the aorta. Velocity magnitude is color coded as shown by the integrated color bar; the pathlines are emitted from two emitter planes.
(MPG)
